# Microstructure and Mechanical Properties of Hypo- and Hypereutectic Cast Mg/Mg_2_Si Composites

**DOI:** 10.3390/ma13163591

**Published:** 2020-08-14

**Authors:** Katarzyna N. Braszczyńska-Malik, Marcin A. Malik

**Affiliations:** Faculty of Production Engineering and Materials Technology, Al, Czestochowa University of Technology, Armii Krajowej19, 42-200 Czestochowa, Poland; marcin.malik@pcz.pl

**Keywords:** magnesium, composite, Mg_2_Si, microstructure, mechanical properties

## Abstract

In this paper, the microstructure and mechanical properties of two magnesium matrix composites—a hypoeutectic with 1.9 wt% Mg_2_Si phase and a hypereutectic with 19 wt% Mg_2_Si compound—were analyzed. The investigated materials were prepared using the gravity casting method. Microstructure analyses of the fabricated composites were carried out by XRD and light microscopy. The tensile and compression strength as well as yield strength of the composites were examined in both uniaxial tensile and compression tests. The microstructure of the hypoeutectic composite was in agreement with the phase diagram and composed of primary Mg dendrites and an Mg–Mg_2_Si eutectic mixture. For the hypereutectic composite, besides the primary Mg_2_Si phase and eutectic mixture, additional magnesium dendrites surrounding the Mg_2_Si compound were observed due to nonequilibrium solidification conditions. The composites exhibited a rise in the examined mechanical properties with an increase in the Mg_2_Si weight fraction and also a higher tensile and compression strength in comparison to the pure magnesium matrix (cast in the same conditions). Additionally, analyses of fracture surfaces of the composites carried out using scanning electron microscopy (SEM + EDX) are presented.

## 1. Introduction

For many years, metal matrix composites (MMCs) have been designed in many different systems in terms of both different metal matrix alloys and various types of reinforcement phase [1,2,3,4,5]. Among them, magnesium matrix composites are very attractive due to the especially low density of the matrix metal. Additionally, thanks to their unique combination of different properties such as exceptional dimensional stability and high damping capacity, specific strength and stiffness, those composites are very attractive in such applications as the aerospace, automobile or electronics industries. Typical ex situ composites, in which reinforcements are introduced from outside to the matrix alloy, comprise the biggest group of magnesium matrix composites. Many different magnesium matrix alloys (from the Mg–Al, Mg–Zn or Mg–rare earth systems) with various reinforced phases (various particles or fibers) such as SiC, C_gr_, TiC, Ti, microspheres etc. have been designed and investigated in recent years [6,7,8,9,10,11,12,13,14].

**Figure 1:**
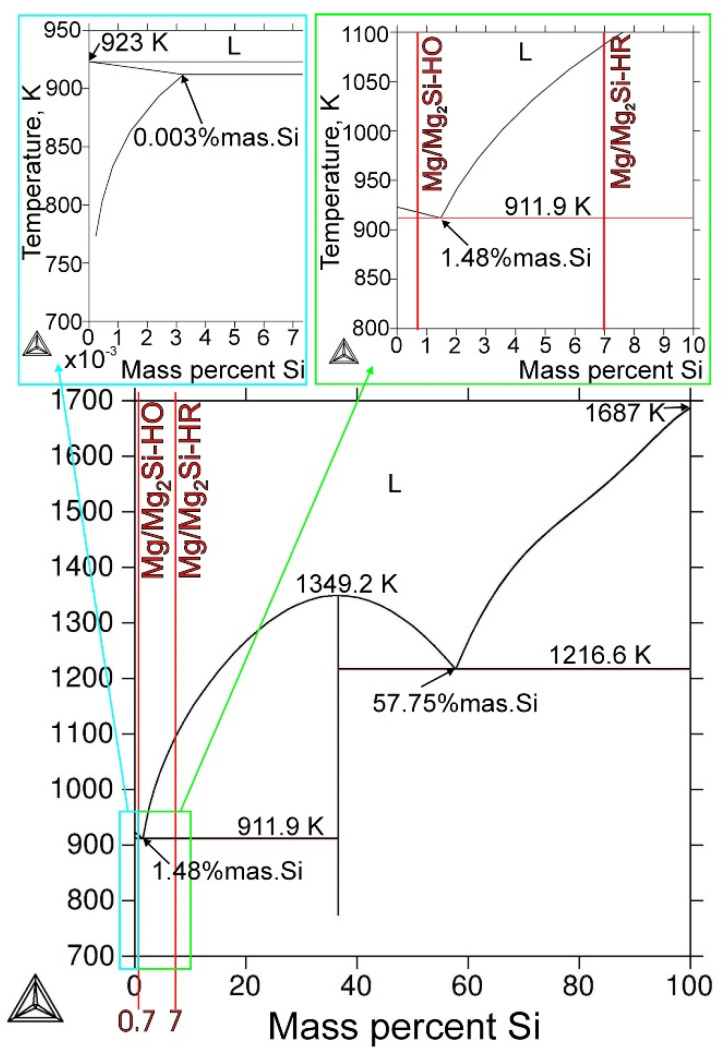
Mg–Si phase diagram (calculated in Thermo-Calc Software; Database: COST2 [48]).

On the other hand, in situ composites constitute a separate group, in which reinforcement is formed inside the matrix. In this group, Mg/Mg_2_Si composites are typical material in which reinforcing the Mg_2_Si phase is created on the inside of the matrix due to the chemical reaction between magnesium and silicon [6,15,16,17,18,19,20,21]. The design of magnesium matrix composites with the Mg_2_Si component is based on the Mg–Si binary phase diagram (presented in Figure 1). According to this diagram, eutectic transformation proceeds at 1.48 wt% silicon, and materials from the Mg–Si system are divided into hypoeutectic, eutectic and hypereutectic. These composites can be fabricated by both powder metallurgy [22,23,24,25,26,27,28] and the casting process [6,29,30,31,32,33,34,35,36,37,38,39,40,41,42,43]. Compared to other methods, casting is a method that can be easily adapted to the required commercial scale of production and is the most economical. Although there is a large difference in the melting temperature of both the used elements, it is possible to dissolve silicon in liquid magnesium, which allows uniform composites with different weight fractions of the Mg_2_Si component to be obtained.

Recently, Mg/Mg_2_Si composites have been intensively investigated due to the number of properties of the Mg_2_Si phase such as low density (1.99 g/cm^3^), a comparatively low thermal expansion coefficient (7.5 × 10^−6^ K^−1^), relatively high Young’s modulus (120 GPa) and high hardness (4.5 × 10^9^ Pa) [17,18,19,20,21,30,31,32,33,34,35,36]. It should be additionally noted that the Mg_2_Si compound is also used as a reinforcing phase of aluminum matrix composites [37,38,39,40,41,42,43] or as a component of magnesium matrix composites with SiC or aluminosilicate microspheres [27,44,45]. However, Mg/Mg_2_Si materials were most often investigated in separate experiments where composites with different weight fractions of silicon (i.e., the Mg_2_Si phase) were analyzed. Pan Y et al. [18] described the microstructure of Mg with 8 wt% Si, whereas in works [17,29], a composite with 5 wt% Si was presented. The effect of the Si content on low frequency damping capacities was investigated for materials with 0.3, 0.8 and 2.3 wt% Si in work [30], but in paper [16], the results for a composite with only 1 wt% Si were presented. Additionally, in many papers, the influence of a third element (such as Bi, Ce, Nd, Y, Sr, Sb) was studied in order to analyze the modification phenomenon of the Mg_2_Si primary phase or eutectic mixture [15,20,21,31,32,33,34,35], but these investigations were also most often performed on materials with one weight fraction of the Mg_2_Si compound. Recently, gradient Mg/Mg_2_Si composites [46] and open cell foams [47] have also been fabricated and studied. Nevertheless, incomplete data concerning the correlation between the fabrication process, microstructure and properties of Mg/Mg_2_Si composites require detailed investigations, especially for future composite design. There are also many divergent results concerning particularly the morphology of the primary Mg_2_Si phase or the influence of the Si (Mg_2_Si) weight fraction on the mechanical properties. In some cases, cubic or polygonal morphology of the Mg_2_Si primary phase was observed [29,30], but in the other works, primary dendrites of this compound were observed in the microstructure of the composites [18,19,36]. There are also poor data describing the mechanical properties of pure Mg with Mg_2_Si composites in as-cast conditions. Hu X.S. et al. [30] reported that a composite with 0.8 wt% Si exhibited the highest tensile strength (152 MPa), whereas the tensile strength decreased for a composite with 2.3 wt% Si (117 MPa). Mirshahi F. et al. [17] obtained an ultimate tensile strength equal to 95 MPa for a composite with 5 wt% Si. Higher values of the ultimate tensile strength were obtained in work [19] for hot extruded (at 623 K) composites with 3, 5 and 7 wt% Si with extrusion ratios of 6:1, 12:1 and 18:1. Unfortunately, the results for as-cast composites at the initial stage (before extrusion) were not given in this work.

In the present paper, particular investigations of the microstructure and mechanical properties of two Mg/Mg_2_Si composites are presented. Hypoeutectic and hypereutectic composites were gravity cast in the same conditions, and the influence of the silicon weight fraction on the properties of the composites tested in both uniaxial tensile and compression tests (also in comparison with pure magnesium) was shown.

## 2. Materials and Methods

Technically pure magnesium and pure silicon in the form of an ingot and powder, respectively, were used in this study. The Mg/Mg_2_Si composites were obtained by the casting method, which involved introducing Si powder into mixed molten magnesium in a steel crucible (with a capacity of about 1.5 kg of molten magnesium) under a protective argon atmosphere. The chemical compositions of the prepared materials were chosen to obtain hypoeutectic and hypereutectic materials according to the phase diagram (Figure 1) calculated in Thermo-Calc Software [48]. The first hypoeutectic material (called Mg/Mg_2_Si–HO in this work) was fabricated with 0.7 wt% silicon, which corresponds to about 1.9 wt% Mg_2_Si in magnesium. The second hypereutectic composite (called Mg/Mg_2_Si–HR in this work) was fabricated with 7 wt% silicon, which corresponds to about 19 wt% Mg_2_Si in magnesium. The prepared composite melts were gravity cast in a cold steel mold, which was designed for magnesium alloys and their composites (with the relatively large riser head and set of gas vents).

The phase compositions of the investigated materials were analyzed by X-ray diffraction (XRD) using a Brucker D8 Advance diffractometer (Bruker Corporation, Billerica, MA, USA) with Cu_Kα_ X-ray radiation. Reflexes from particular phases were identified according to ICDD PDF-4+ cards [49]. The specimens for the microstructure investigations were prepared by standard metallographic procedures. To reveal the microstructure, the samples were etched in a 1% solution of HNO_3_ in C_2_H_5_OH for about 60 s. The microstructures were observed with an Olympus GX51 light microscope (LM) (Olumpus, Tokyo, Japan) with differential interface contrast (DIC).

Mechanical properties tests of the composites were carried out according to relevant ASTM standards on a Zwick/Roell Z100 machine (Zwick Roell Group, Ulm, Germany) with a strain rate of 0.01 mm/s. The performed mechanical tests included experimental determination of the ultimate tensile strength (UTS) and yield strength (TYS) on standard rodlike samples with a diameter of 8 mm in a uniaxial tensile test. Compression strength (CS) and yield strength under compression (YS) were determined in the uniaxial compression test on samples with a diameter of 8 mm and length of 12 mm. Both tests were carried out at room temperature. For comparison, the same mechanical tests were performed for the used technically pure magnesium (cast in the same conditions in the same mold as the fabricated composites). For each material, three samples were tested. In addition, the fracture surfaces of the investigated composites after uniaxial tensile testing were observed by a JEOL JSM-6610LV scanning electron microscope (SEM) (JEOL Ltd., Tokyo, Japan) with an energy dispersive X-ray spectrometer (EDX).

## 3. Results and Discussion

**Figure 2:**
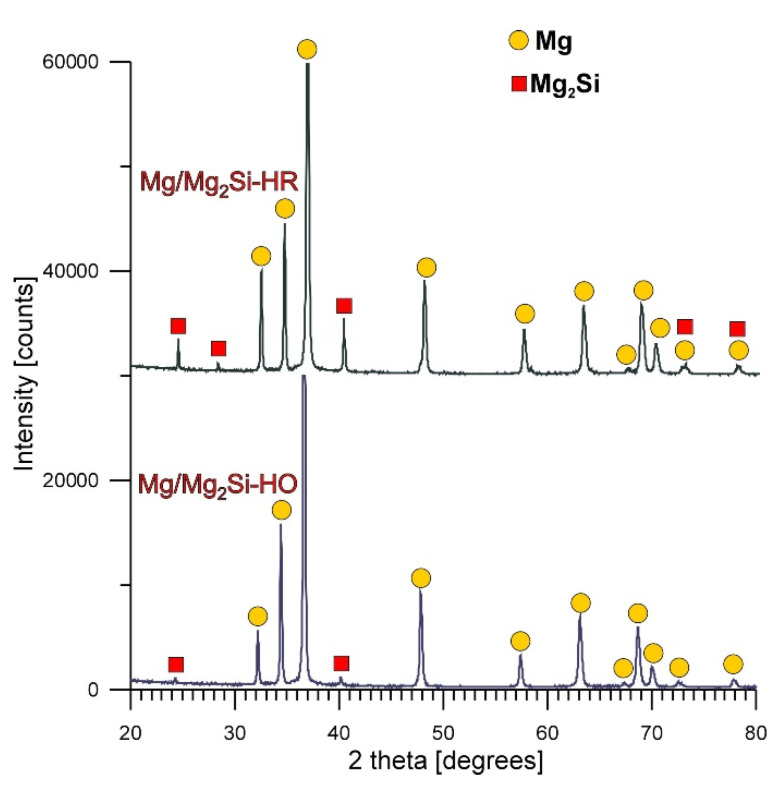
X-ray diffraction patterns of Mg/Mg_2_Si–HO and Mg/Mg_2_Si–HR composites.

Figure 2 shows the X-ray diffraction micrographs for the Mg/Mg_2_Si–HO and Mg/Mg_2_Si–HR fabricated composites. It confirmed that both materials were composed of Mg and Mg_2_Si phases. Additionally, the comparison of the X-ray patterns obtained for both materials revealed a distinct increase in the reflex intensity from the Mg_2_Si phase in the Mg/Mg_2_Si–HR rather than in the Mg/Mg_2_Si–HO composite, which confirmed the rise in the volume fraction of this structural constituent in the material with the higher weight fraction of silicon. It should also be noted that reflexes from pure silicon were not registered, confirming that all the silicon was introduced into the molten magnesium and created the Mg_2_Si phase.

**Figure 3:**
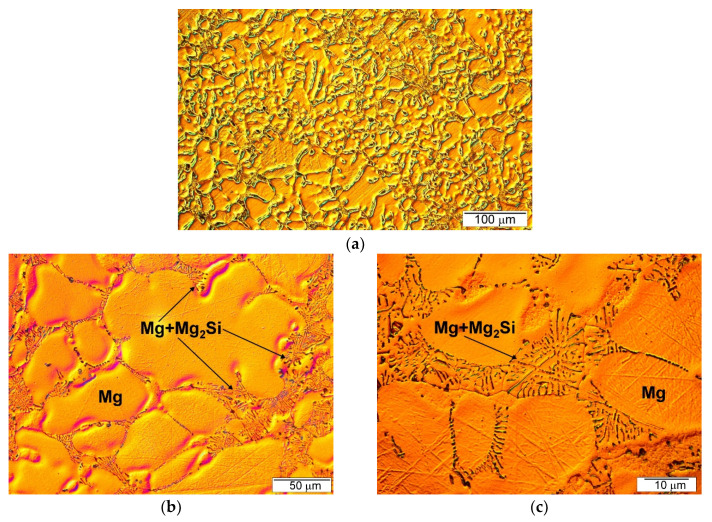
Microstructure of Mg/Mg_2_Si–HO composite (**a**–**c**) three micrographs taken at different magnifications for Mg/Mg2Si–HO.

**Figure 4:**
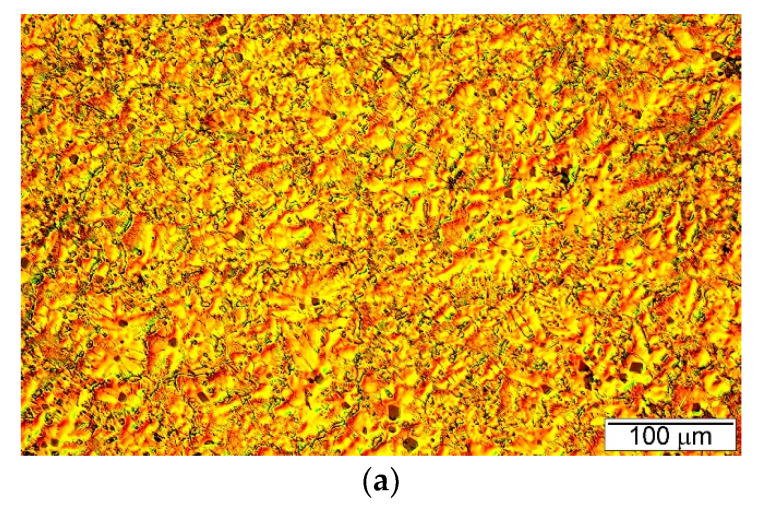

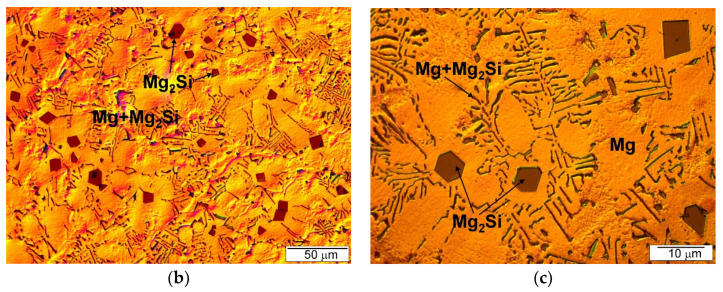
Microstructure of Mg/Mg_2_Si–HR composite (**a**–**c**) shows three micrographs taken at different magnifications for Mg/Mg_2_Si–HR.

**Figure 5:**
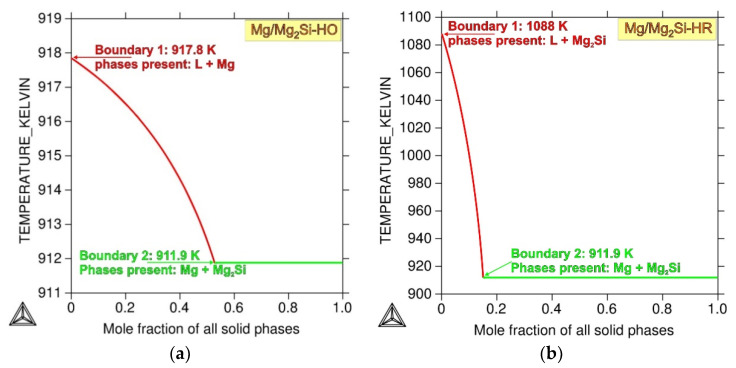
Sheil solidification simulation for Mg/Mg_2_Si–HO (**a**) and Mg/Mg_2_Si–HR (**b**) composites (calculated in Thermo-Calc Software [48]).

Typical microstructure micrographs of both the fabricated composites are presented in Figure 3 and Figure 4. It should be noted that according to the phase diagram (Figure 1), the maximal solid-state solubility of Si in Mg was equal to only 0.003 wt% at the temperature of eutectic transformation (911.9 K). According to this phase diagram, the α (Mg) silicon in the magnesium solid solution formed in equilibrium solidification conditions. However, it could be accepted that due to the very limited solubility of silicon in magnesium and fast solidification of fabricated composites in cold steel molds, which cause strong deviation from equilibrium solidification conditions, the primary crystals solidifying from the liquid were practically pure magnesium. The comparison of Figure 3a and 4a (made at the same magnification) shows that the silicon weight fraction contributed to the refinement of the microstructure of the obtained materials. The microstructure of the Mg/Mg_2_Si–HO composite (Figure 3) was composed of the dendrite of primary magnesium and Mg + Mg_2_Si eutectic, located in the interdendritic spaces. This microstructure is in agreement with the solidification curves calculated according to the Sheil model (in Thermo-Calc Software), which assumes a total lack of diffusion in the solid state and complete mixing in the liquid state (Figure 5a). Although real solidification is generally expected to be between the Scheil and equilibrium solidification conditions, in the case of the investigated Mg–Si system, the curves calculated according the Scheil model and in equilibrium conditions were practically the same due to the negligible solubility of silicon in magnesium. The obtained microstructure of the Mg/Mg_2_Si–HO composite corresponded to the sequence of the solidification curve according to which solidification began with the formation of Mg crystals and ended with the eutectic transformation of Mg + Mg_2_Si. It should also be noted that the eutectic formed in the investigated materials had irregular morphology—typical for a faceted–nonfaceted eutectic.

The microstructure of the Mg/Mg_2_Si–HR composite presented in Figure 4 is characterized by the presence of the primary Mg_2_Si phase. The primary Mg_2_Si phase has a regular polygonal morphology (typical for faceted crystals) and assumes the shape of a hexahedron, octahedron or tetrakaidecahedron. This morphology of primary Mg_2_Si crystals has been observed in different composites reinforced with this compound; however, it was also mentioned in the Introduction Section that dendritic morphology (with visible dendrite arms) of the primary Mg_2_Si phase was also observed. Mirshahi R. et al. [29] investigated a Mg −5 wt% Si composite and concluded that the morphology of the Mg_2_Si phase depends on cooling rates in the range between 20 to 1.2 K/s. They observed that at high cooling rates, Mg_2_Si formed with polygonal morphology, while at lower cooling rates Mg_2_Si precipitated dendritically. These results are in contradiction to the results of Pan X et al. [18], which revealed dendrites of the primary Mg_2_Si phase in composites with 8 wt% Si cast in a steel mold. On the other hand, in work [19], dendrites of the Mg_2_Si phase were observed in composites cast in an iron mold preheated to 473 K. In contrast, polygonal Mg_2_Si crystals were disclosed in work [30] in composites with 2.3 wt% Si cast in a steel mold preheated to 673 K. In our previous study [36], typical dendrites of Mg_2_Si were identified in a composite based on an AM50 magnesium matrix alloy with 9.9 wt% Mg_2_Si cast in a cold steel mold. In the presented study, dendritic growth of the Mg_2_Si crystals was not observed. The observed differences in the primary Mg_2_Si phase morphology in various works could be the effect of not only the cooling rate but also the chemical composition of the composite (and the presence of further elements or impurities) and super-cooling during solidification (which also depends on the casting temperature). These factors need very detailed studies especially in the context of impurities. It is most likely that a very small amount of surface-active third elements influenced the phase morphology, similar to the aluminum–silicon system (in which a content of up to 9 ppm phosphorus causes changes in the microstructure of hypereutectic alloys).

**Figure 6:**
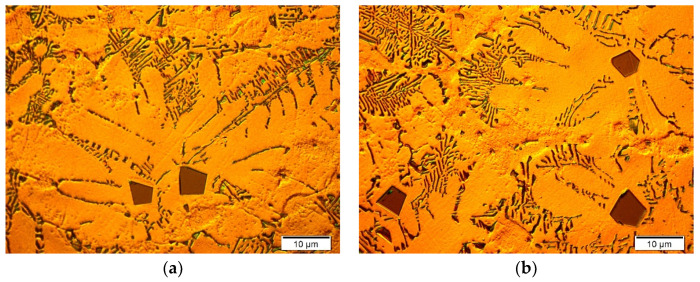
Micrographs of Mg/Mg_2_Si–HR composite microstructure presenting magnesium dendrites surrounding primary Mg_2_Si crystals. (**a**,**b**) shows two micrographs taken from different areas of Mg/Mg2Si–HR.

The formation of the primary Mg_2_Si phase in the Mg/Mg_2_Si–HR composite is in agreement with both the phase diagram (Figure 1) and the solidification curves calculated according to the Sheil model shown in Figure 5b. The sequence of solidification according to these curves described the crystallization of the Mg_2_Si phase at the beginning and eutectic transformation at the end. The obtained microstructure of the Mg/Mg_2_Si–HR composite had, though, an additional structural constituent in the form of magnesium dendrites surrounding the primary Mg_2_Si crystals. These dendrites of magnesium arose due to local fluctuation of the chemical composition of the composite during nonequilibrium solidification. The Mg_2_Si crystals, formed as the first, needed a relatively high amount of silicon, which caused impoverishment of this element in the surrounding liquid. On one hand, this impoverishment blocked the growth of primary Mg_2_Si crystals, and on the other hand, there were advantageous conditions for the nucleation of magnesium. Below the temperature of 923 K, the magnesium dendrites nucleated in these regions and quickly grew. At the end of solidification, the last part of the liquid with eutectic composition formed an Mg + Mg_2_Si eutectic mixture. Mg dendrites are visible in Figure 4 and, additionally, in the micrographs presented in Figure 6. The dendrites were of different sizes and some of them also had secondary arms, visible especially in Figure 4a and Figure 6, which can indicate local fluctuation of the chemical composition in the liquid. The distribution of magnesium dendrites was caused by the distribution of elements in the liquid during nonequilibrium solidification conditions, but the presence of the primary Mg_2_Si phase inside all the dendrites could also suggest heterogeneous nucleation of magnesium on the Mg_2_Si crystals. It is well known that one of the necessary conditions for heterogeneous nucleation is the formation of a low energy interface between the nucleus and substrate, i.e., a coherent (or at least a semicoherent) interface with a small lattice misfit along the interface. According to typical crystallographic calculations in the main directions of the basal planes, Mg and Mg_2_Si exhibited an interatomic spacing misfit of less than 0.1, i.e., the critical value for a coherent interface.

**Figure 7:**
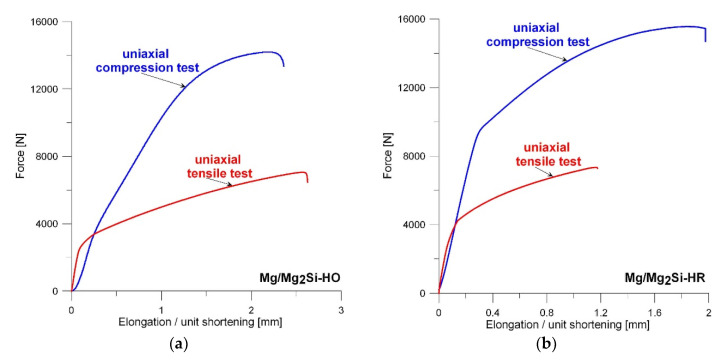
Representative tension and compression curves for the Mg/Mg_2_Si–HO (**a**) and Mg/Mg_2_Si–HR composites (**b**).

**Figure 8:**
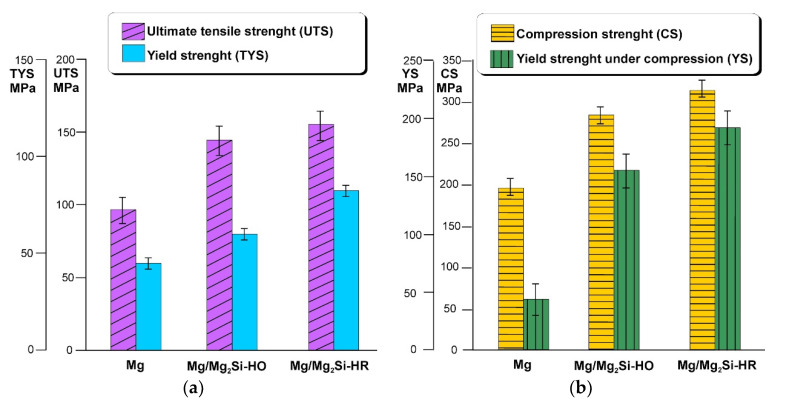
Average values of ultimate tensile strength (UTS) and yield strength (TYS) (**a**), compression strength (CS) and yield strength under compression (YS) (**b**) for Mg/Mg_2_Si–HO and Mg/Mg_2_Si–HR composites comprised with technically pure magnesium (with scatter of results).

The significant differences in the microstructure of both the investigated composites described above, resulting from the weight fraction of silicon, had an influence on the obtained values of the mechanical properties of the Mg/Mg_2_Si–HO and Mg/Mg_2_Si–HR materials. The representative tension and compression curves for the Mg/Mg_2_Si–HO and Mg/Mg_2_Si–HR composites are shown in Figure 7. Figure 8a presents the ultimate tensile strength (UTS) and yield strength (TYS) obtained in the uniaxial tensile test for both composites and compared with those obtained for technically pure magnesium cast in the same conditions. The analogical results presenting the compression strength (CS) and yield strength (YS) values obtained in the uniaxial compression test are shown in Figure 8b. Both the fabricated composites exhibited higher mechanical properties than technically pure magnesium. The obtained results of the ultimate tensile strength of both the investigated composites were also higher than those presented by Mirshahi F. et al. [17] for an unmodified composite with 5 wt% silicon and a modified one with 0.5 wt% Bi, where in both materials, the obtained ultimate tensile strength was about 100 MPa. On the other hand, both in the uniaxial tensile test and uniaxial compression test, the highest values were obtained by the Mg/Mg_2_Si–HR composite rather than by the Mg/Mg_2_Si–HO composite, which is in contradiction to the results obtained by Hu X.S. et al. [30], where the tensile strength of the composites decreased with a rise in the weight fraction of silicon. For the investigated composites, the ultimate tensile strength and compression strength of the Mg/Mg_2_Si–HO composite at room temperature were 145 and 283 MPa, respectively, while the highest UTS was 155 MPa and CS was 315 obtained by the Mg/Mg_2_Si–HR composite. These values are comparable with the results obtained by gravity cast magnesium alloys, for example AM50 or AME505 [50]. On the other hand, the presented composites had a yield strength of more than twice as high, especially under compression. The Mg/Mg_2_Si–HR composite exhibited a yield strength of more than twice high under compression as the highly resistant AME505 magnesium alloy [50] and significantly higher than the one obtained by the gradient composite (about 200 MPa) [46].

**Figure 9:**
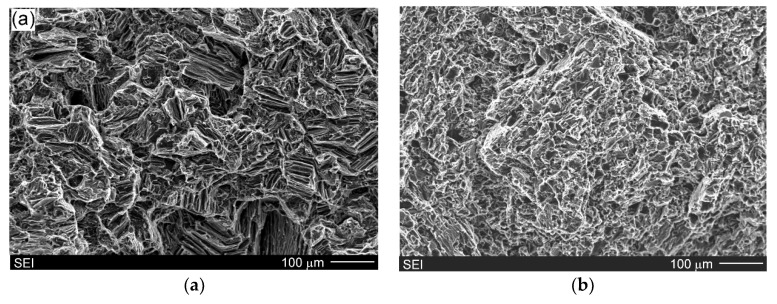
SEM micrographs of fracture surface of Mg/Mg_2_Si–HO (**a**) and Mg/Mg_2_Si–HR (**b**) composites (after uniaxial tensile test).

**Figure 10:**
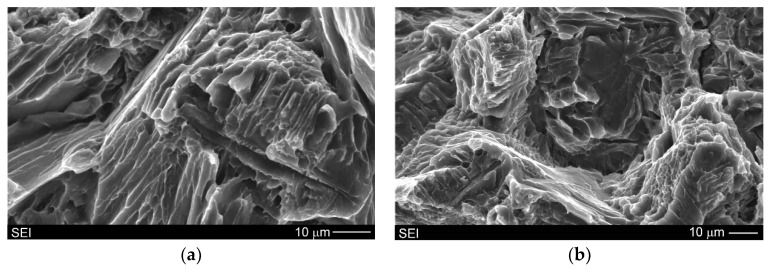
SEM micrographs of Mg/Mg_2_Si–HO composite fracture surfaces illustrating cracking through Mg + Mg_2_Si eutectic (after uniaxial tensile test) ((**a**,**b**) taken from different areas of fracture surfaces of the same material).

Figure 9a,b show SEM micrographs of fracture surfaces after the uniaxial tensile test of the Mg/Mg_2_Si–HO and Mg/Mg_2_Si–HR composites, respectively. The investigated materials exhibited different fracture surfaces. Refinement of the composite microstructures with the increase in the weight fraction of the Mg_2_Si compound observed during the comparison of Figure 4a and Figure 5a is also visible when comparing Figure 9a,b (produced at the same magnification). The fracture surface of the Mg/Mg_2_Si–HO composite was characterized by cleavage steps, which are typical for magnesium. The failure of magnesium is usually brittle through cleavage or quasi-cleavage due to the hexagonal closed packed structure. Higher magnifications used during fracture surface observations (Figure 10) also revealed cracking through the Mg + Mg_2_Si eutectic.

**Figure 11:**
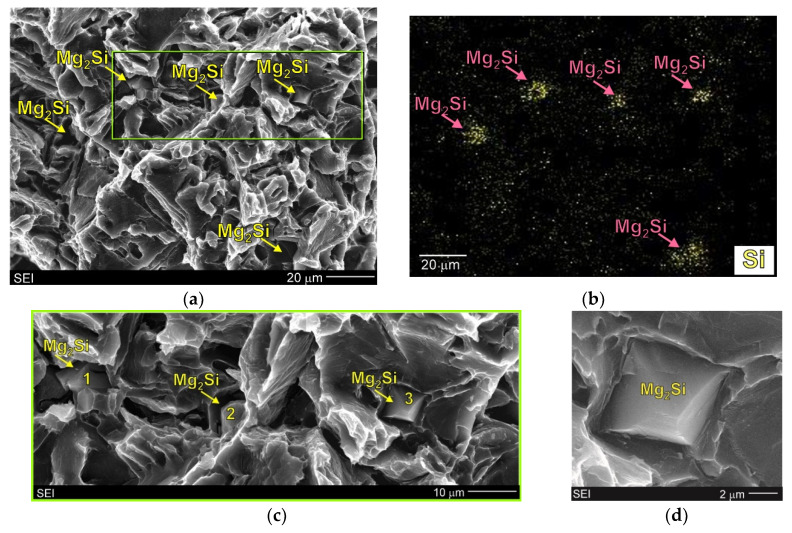
SEM micrographs of Mg/Mg_2_Si–HR composite fracture surfaces (after uniaxial tensile test) (**a**) with adequate silicon surface distribution (EDX) (**b**), higher magnification of area in green frame (**c**) and higher magnification of the Mg_2_Si phase designated as 3 in (**c**) micrograph (**d**).

**Figure 12:**
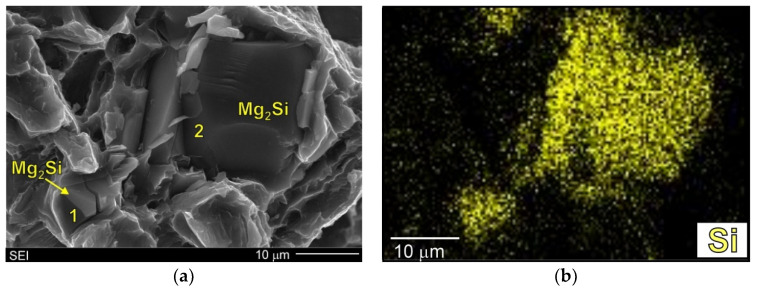
SEM micrographs of Mg/Mg_2_Si–HR composite of fracture surfaces (after uniaxial tensile test) (**a**) with adequate silicon surface distribution (EDX) (**b**).

The fracture surface of the Mg/Mg_2_Si–HR composite was more developed. Figure 11 and Figure 12 show SEM micrographs of the fracture surfaces of this composite at higher magnification. The primary Mg_2_Si phase was clearly visible on the fracture surface. In Figure 11 and Figure 12, the surface distributions of the main element were also added (11b, 12b). Although the energy dispersive X-ray spectrometry (EDX) results from the fracture surfaces were burdened with errors (especially quantitatively), they confirmed the presence of primary Mg_2_Si crystals in the obtained micrographs. It should also be noted that the particles of the primary Mg_2_Si phase were cracked (and exhibited intrinsic brittleness). This effect was also observed in work [17] for the composite with 5 wt% silicon where particle decohesion was also concluded. Nonetheless, detailed analyses of the fracture surfaces of the material investigated in the present study did not indicate this mechanism in the Mg/Mg_2_Si–HR composite. The micrographs presented in Figure 11c,d and Figure 12 show that the cracking process proceeded through the Mg_2_Si with the propagation of secondary cracks. All the Mg_2_Si phases designated as 1–3 in Figure 11c and as 1–2 in Figure 12a had visible effects of brittle cracking. The presented SEM results also indicate that primary Mg_2_Si crystals and the surrounding magnesium phase were strongly connected, which could also be an additional argument for the heterogeneous nucleation of magnesium dendrites on the Mg_2_Si phase. The magnesium dendrite surrounding the Mg_2_Si cracked particle (described as 3) is especially visible in Figure 11.

## 4. Conclusions

In the presented paper, two hypo- and hypereutectic Mg/Mg_2_Si composites were studied. The main conclusions drawn are as follows:

1 Magnesium matrix composites with 1.9 and 19 wt% Mg_2_Si phase were successfully fabricated by the casting method.
2 The microstructure of the material with 1.9 wt% Mg_2_Si consisted of primary magnesium dendrites and an Mg + Mg_2_Si eutectic mixture, whereas the composite with 19 wt% Mg_2_Si exhibited a primary polygonal Mg_2_Si compound surrounded by magnesium dendrites and eutectic.
3 The composites exhibited a rise in tensile and yield strength in both the tensile and compression tests with an increase in the weight fraction of the Mg_2_Si phase.
4 The fracture surface observations revealed that during the uniaxial tensile test, the cracking process of the fabricated composites proceeded through all structural constituents.
